# Predictors of severe stenosis at invasive coronary angiography in patients with normal myocardial perfusion imaging

**DOI:** 10.1007/s12471-018-1091-7

**Published:** 2018-03-02

**Authors:** S. Yokota, J. P. Ottervanger, M. Mouden, M. J. de Boer, P. L. Jager, J. R. Timmer

**Affiliations:** 1 Department of Cardiology, Isala Hospital, Zwolle, The Netherlands; 2 Department of Cardiology, Radboud University Medical Center, Nijmegen, The Netherlands; 3 Department of Nuclear Medicine, Isala Hospital, Zwolle, The Netherlands

**Keywords:** Single photon emission computed tomography, Myocardial perfusion imaging, Coronary artery disease, Gender

## Abstract

**Purpose:**

Normal myocardial perfusion imaging (MPI) is associated with excellent prognosis. However, in patients with persisting symptoms, it may be difficult to determine the patients in whom invasive angiography is justified to rule out false negative MPI. We evaluated predictors for severe stenosis at invasive angiography in patients with persisting symptoms after normal MPI.

**Methods:**

229 consecutive patients with normal MPI, without previous bypass surgery, underwent invasive angiography within 6 months. Older age was defined as >65 years. Multivariable analyses were performed to adjust for differences in baseline variables.

**Results:**

Mean age was 62 ± 11 years, 48% were women. Severe stenosis was observed in 34%, and of these patients 60% had single-vessel disease (not left main coronary artery disease). After adjusting for several variables, including diabetes, smoking status, hypertension and hypercholesterolaemia, predictors of severe stenosis were male gender, odds ratio (OR) 2.7 (95% confidence interval (CI) 1.5–4.9), older age, OR 1.9 (95% CI 1.02–3.54) previous PCI, OR 2.0 (95% CI 1.0–4.3) and typical angina, OR 2.5 (95% CI 1.4–4.6).

**Conclusions:**

Increasing age, male gender, previous PCI and typical symptoms are predictors of severe stenosis at invasive coronary angiography in patients with normal MPI. The majority of these patients have single-vessel disease.

## Introduction

Myocardial perfusion imaging (MPI) using single photon emission computed tomography (SPECT) is a frequently used non-invasive modality in patients with suspected angina. A normal MPI is associated with an excellent prognosis [1, 2]. Although the diagnostic accuracy of MPI is good [3], the possibility of a false negative test should be considered, particularly in patients with persisting symptoms [4]. Since invasive coronary angiography (ICA) is still the gold standard for ruling out obstructive coronary disease, it can be considered in patients with normal MPI with persisting symptoms. In daily clinical practice, it remains, however, a challenge to determine in which patients ICA is justified. There are only few studies concerning predictors of abnormalities in patients with normal MPI, and many are hampered by a small sample size [5, 6].

The aim of our study was to assess independent predictors of severe coronary stenosis as detected with ICA in patients with normal MPI and persisting symptoms.

## Materials and methods

### Study population

We performed a retrospective analysis of all 11,402 consecutive patients who underwent MPI using 99mTc-Tetrofosmin SPECT in the Isala Hospital, in Zwolle, the Netherlands between January 2006 and December 31^st^, 2009. Subsequent ICA within six months was performed in 1,602 (14%) patients, including 256 stable patients with normal MPI and persisting or worsening symptoms. After excluding 27 patients with a history of coronary bypass surgery, we analysed the remaining 229 patients in our current study. We described the methods of the study in an earlier article [7]. We performed the study in accordance with the Declaration of Helsinki.

### Clinical information

At the time of MPI, all patients completed a questionnaire regarding demographic information, prior medical history, cardiac risk factors and current medication use. Furthermore, information regarding patient age, gender, weight, height, blood pressure, heart rate and symptoms were prospectively obtained by a nurse.

Typical angina was defined as the presence of substernal chest pain or discomfort that was provoked by exertion or emotional stress and relieved by rest and/or nitroglycerin [8].

Left ventricle ejection fraction (LVEF) and dimensions were assessed by echocardiography. LVEF <50% was considered abnormal. End diastolic left ventricular internal diameter was defined as dilated if the value was ≥59 mm (for men) or ≥53 mm (for women).

### Classification of coronary disease

Invasive angiography was performed with the Judkins or radial approach. Two experienced interventionalists blinded to MPI results retrospectively re-interpreted all angiograms visually. A coronary stenosis of ≥70% was considered to be severe for the left anterior descending artery (LAD), left circumflex artery (LCx) and right coronary artery (RCA). Severe left main coronary artery disease (LMCAD) was defined as >50% diameter stenosis [9]. Patients were categorised as having LMCAD (both isolated and non-isolated), single-vessel disease without LMCAD, two-vessel disease without LMCAD and three-vessel disease without LMCAD. Performance of fractional flow reserve (FFR) was at the discretion of the operator. We used an FFR cut-off value of ≤0.80.

### SPECT MPI data acquisition and analysis

All patients underwent a 1-day stress 99mTc-Tetrofosmin MPI protocol. Owing to logistic reasons we routinely use adenosine for stress unless contraindicated. We used adenosine in 219 patients, dobutamine in 3 patients and physical exercise (bicycle) in 7 patients. All MPI scans were analysed by experienced readers as previously reported [7]. Transient ischaemic left ventricular dilatation was defined as abnormal if the value was >1.36 for adenosine stress [10]. Scans were considered normal if perfusion was assessed to be homogenous throughout the myocardium and summed stress score was ≤3 [11]. Two readers retrospectively re-analysed all scans to unmask any potential abnormality that was initially missed.

### Follow-up

Survival status was evaluated in August 2015 using the ‘Gemeentelijke Basis Administratie’ system, a decentralised Dutch population registration system that contains information about all inhabitants of the Netherlands. Since no data are erased from this system, new data, such as death and emigration, are registered in the register. The data were considered right-censored if patients were still alive at the time of evaluation. In addition, data on symptom status were obtained from medical chart records for patients who underwent revascularisation to assess the impact from the coronary intervention.

### Statistical analysis

Statistical analysis to compare baseline characteristics was performed with a Chi-Square test and one-way analysis of variance (ANOVA) as available in SPSS software (version 20 for Windows; SPSS Inc., Chicago, Illinois, USA). Comparison of continuous data between both groups was performed using the two-sided student’s t‑test. Quantitative variables were expressed as mean ± SD and categorical variables as frequencies, or percentages. Logistic regression analyses were performed to assess independent predictors of severe stenosis at angiography. The Kaplan-Meier method was used for univariate survival analysis. Cox proportional hazards regression model was used to assess whether abnormal findings at angiography were independent predictors of mortality. *P*-values of <0.05 were considered statistically significant.

## Results

Mean age of the 229 patients was 62 ± 11 years, 48% were women and mean body mass index (BMI) was 28 ± 5 kg/m^2^. Hypertension and hypercholesterolaemia were found in more than 50% of patients. A total of 20% of patients had a previous percutaneous coronary intervention (PCI). Aspirin, beta blockers and statins were prescribed to the majority of patients before referral for ICA. In 52% of the patients, the symptoms were interpreted as typical angina; there was no difference between men (51%) and women (53%, *p* = 0.82). We observed abnormal LVEF in 78 patients (34%).

### Invasive angiography

**Fig. 1:**
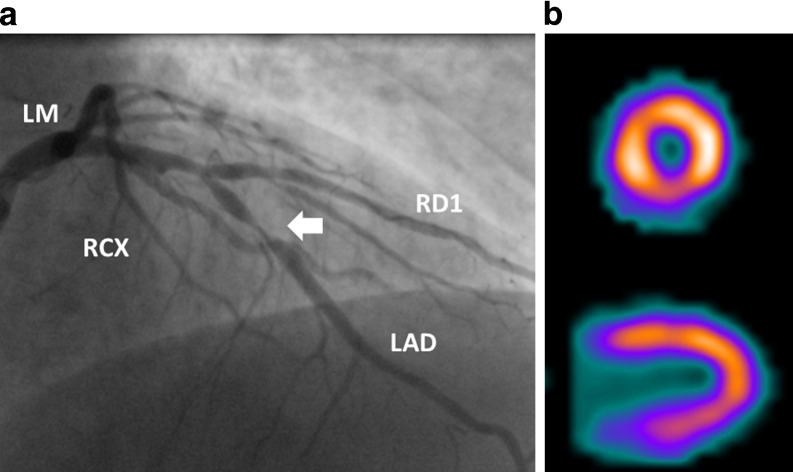
Coronary angiogram showing significant stenosis (arrow) in mid-segment of LAD (**a**) in a patient with persisting angina and normal SPECT MPI (**b**). (*LAD* left anterior descending artery, *LM* left main coronary artery, *RCX* right circumflex artery, *RD1* diagonal branch)

Mean time between MPI and ICA was 66 ± 44 days (range 2–182 days). A total of 78 patients (34%) had severe coronary stenosis. In 9 patients, severity was confirmed by measurement of FFR. Of the 78 patients with severe stenosis, 47 patients (60%) had single-vessel disease (not the left main coronary artery), 18 patients (23%) had two-vessel disease, whereas 13 patients (17%) had LMCAD and/or three-vessel disease. There were no patients with isolated stenosis of the left main coronary artery. An example of a normal SPECT MPI in a patient with a severe stenosis is displayed in Fig. 1. Of the 47 patients with one-vessel disease, the stenosis was located in the LAD in 45%, in the RCA in 30% and in the LCX in 26%.

Severe stenosis was more common in men (64%) and in patients with older age, a history of PCI, and typical anginal symptoms (Tab. 1). The prevalence rates of abnormal LVEF, dilatation of left ventricle, atrial fibrillation or left bundle branch block, and symptoms during pharmacologic stress were not significantly higher in patients with severe stenosis compared to those with non-severe stenosis. Transient ischaemic left ventricular dilatation was only observed in 2 patients (3%, *p* = 0.11%), both with severe coronary stenosis.

**Table 1:** Comparison between severe and non-severe coronary artery disease, as assessed by invasive coronary angiography in patients with normal SPECT

Total Cohort *N* = 229	Severe stenosis (*N* = 78)	No severe stenosis (*N* = 151)	*P*-value
Age (years)	65 ± 10	61 ± 11	0.002^*^
Length (cm)	173 ± 7	172 ± 10	0.44
Weight (kg)	83 ± 15	84 ± 16	0.61
BMI (kg/m^2^)	27 ± 4	28 ± 5	0.25
Male gender	50 (64%)	69 (46%)	0.008^*^
Smoking	18 (23%)	41 (27%)	0.50
Hypertension	53 (68%)	86 (57%)	0.11
Hypercholesterolaemia	57 (73%)	98 (65%)	0.21
Diabetes	18 (23%)	33 (22%)	0.84
Previous PCI	21 (27%)	24 (16%)	0.047^*^
LV dilatation	15 (19%)	23 (15%)	0.27
Abnormal LVEF	14 (18%)	27 (18%)	0.38
Sinus rhythm	74 (95%)	135 (89%)	0.17
Atrial fibrillation	4 (5%)	15 (10%)	0.22
Left bundle branch block	2 (3%)	6 (4%)	0.58
*Medication Use*			
Aspirin	70 (90%)	95 (63%)	<0.001^*^
Beta blocker	65 (83%)	110 (73%)	0.08
Calcium-channel blocker	22 (28%)	35 (23%)	0.40
Statin	53 (68%)	88 (58%)	0.15
Nitrate	39 (50%)	47 (31%)	0.005^*^
Typical angina	52 (67%)	67 (44%)	0.001
*Severity of complaints* (CCS-classification)			0.78
Class I–II	44 (56%)	103 (68%)	
Class III–IV	34 (44%)	48 (32%)	

*BMI* body mass index, *LVEF* left ventricular ejection fraction, *PCI* percutaneous coronary intervention, *SPECT* single photon emission computed tomography

^*^Denotes statistical significance. Adjusted for differences in the other variables

### Independent predictors of severe stenosis

To assess independent predictors of severe stenosis, multivariate analyses were performed. After adjusting for several variables, including diabetes, smoking, hypertension and hypercholesterolaemia, only male gender, odds ratio (OR) 2.7 (95% confidence interval (CI) 1.5–4.9), older age, OR 1.9 (95% CI 1.02–3.54) and typical angina, OR 2.5 (95% CI 1.4–4.6) were predictors of significant coronary stenosis. Results of multivariable analyses are summarised in Tab. 2.

**Table 2:** Predictors of severe coronary artery disease, as assessed by invasive coronary angiography in patients with normal SPECT, after multivariate analyses

Variable	OR	95% CI	*P*-value
Age ≥65 years	1.9	1.0–3.6	0.04^*^
Male gender	2.7	1.4–4.9	<0.01^*^
Diabetes	1.1	0.6–2.3	0.76
Hypertension	1.6	0.8–3.0	0.19
Current smoker	1.0	0.5–2.1	0.99
Hypercholesterolaemia	1.3	0.7–2.5	0.40
Typical angina	2.5	1.4–4.6	<0.01^*^
Previous PCI	2.0	1.0–4.3	<0.05^*^

*CI* confidence interval, *OR* odds ratio, *PCI* percutaneous coronary intervention, *SPECT* single photon emission computed tomography

^*^Denotes statistical significance. Adjusted for differences in the other variables

### Follow-up

Total follow-up duration in survivors was 7.0 years (range 5–9 years, SD 1.1 years). Coronary revascularisation was performed in 70 patients (89%), in 47 patients (60%) by PCI and in 23 patients (30%) by bypass surgery. Among the 70 patients who underwent coronary revascularisation, 49 patients (70%) were free from symptoms, while 16 patients had unchanged persistent chest symptoms (23%). Data on symptom status post intervention were lacking for the remaining 5 patients (7%).

During follow-up, a total of 31 patients died (14%). Mean age of patients who died was higher than of those who survived (71.1 years SD 7.9 vs 60.9 years SD 10.2; *p* < 0.001). After univariate analyses, long-term mortality was 16.7% in patients with severe stenosis and 11.9% in patients without severe stenosis (*p* = 0.32). Also, after adjusting for differences in age, gender and typical angina symptoms, severe stenosis demonstrated by ICA was still not associated with increased mortality, OR 1.0 (95% CI 0.47–2.1).

## Discussion

In our observational study, increasing age, male gender and typical angina symptoms are predictors of severe stenosis demonstrated by ICA in patients with normal MPI. The severe stenoses in our patients were not associated with increased long-term mortality.

MPI is well-validated and has proven value in identifying patients at high risk of a serious cardiac event, whereas a normal MPI study confers a benign prognosis with a low annual serious cardiac event rate of 0.6% per year [12]. However, there can always be concern that MPI has missed high-risk coronary disease as in patients with balanced ischaemia due to flow-limiting three-vessel disease or stenosis of the left main coronary artery. Although balanced ischaemia was considered an important reason for false negative MPI in previous studies [13, 14], the majority of patients with severe stenosis in our study had single-vessel disease. This suggests that balanced ischaemia may be less important as a cause of false negative MPI than previously thought. Various explanations for discordant findings have been suggested: false negative SPECT may be due to inadequate vasodilatation during stress, for example due to recent caffeine intake in an adenosine stress test or inadequate exercise in a physical stress test. But FFR, which is increasingly considered the new gold standard, can result in incorrect diagnoses as well. Reasons may be insufficient hyperaemia, guiding catheter related pitfall, electrical drift, diffuse disease rather than focal stenosis, small perfusion territory, severe microvascular disease, abundant collaterals and severe left ventricular hypertrophy [15, 16].

Since false negative findings may occur in every diagnostic test, it is important to know which patients have the highest risk of a false negative test, and who may benefit from additional (invasive) testing. Accurate detection of false negative findings may lead to initiation of appropriate medical treatment that may improve outcome. Only three studies have assessed predictors of severe stenosis in patients with normal MPI (Tab. 3). The largest study was performed in the US as recently reported [17]. As in our study, they found that increasing age, male gender and typical anginal symptoms are predictors of severe coronary stenosis. Ghadri et al. demonstrated in a small study that a very high coronary calcium score is a predictor of severe coronary stenosis in patients with normal MPI, but they did not assess other potential risk factors [18].

**Table 3:** Predictors of severe stenosis with invasive coronary angiography in patients with normal SPECT MPI in previous studies

Study	Year of publication	Total amount of patients	FFR	Predictors	Comments
Fujimoto et al. [6]	2006	58	0%	Age, hypertension, typical angina	Unstable angina included
Sharma et al. [5]	2010	76	0%	Diabetes, ischaemic ECG response	Only patients with combination of diabetes and ischaemic ECG changes tended to have obstructive CAD
Nakanishi et al. [17]	2015	580	0%	Age, male gender, typical angina, SSS, TID ratio, EF change, TPD	

*CAC* coronary calcium score, *CAD* coronary artery disease, *ECG* electrocardiography, *EF* ejection fraction, *SSS* summed stress score, *SPECT MPI* single photon emission computed tomography myocardial perfusion imaging, *TID* transient ischaemic dilatation, *TPD* total perfusion defect

Stable angina is the most common type of angina, and it typically occurs with exertion and relieves with rest. Our study highlights the importance of adequate history taking in the evaluation of chest pain. This was also observed in previous studies. Cooke and colleagues examined the description of pain in 65 patients with normal angiograms compared to 65 patients with significant coronary stenosis [19]. The presence of two out of three specific symptoms was noted in their study in 85% of patients with significant disease while it was only observed in 26% of those with normal angiograms. Also, other studies showed the importance of medical history for both diagnosis and prognosis in patients with suspected angina [20]. Similarly, in patients referred for coronary computed tomography angiography, the importance of symptoms was demonstrated [21].

Our study was too small to assess whether typical symptoms predict severe stenosis in men and women. In general, women have different symptoms, which are more frequently not recognised as angina [22]. They often experience symptoms such as nausea, shortness of breath, abdominal pain or extreme fatigue, with or without chest pain. It is also more difficult to demonstrate microvascular disease (which is more common in women) with ICA.

We found that ICA in older patients with normal MPI more commonly demonstrates stenosis. First, we should be aware that the prevalence of abnormal ICA is high, even in asymptomatic older patients. Second, it remains to be seen whether the symptoms of older patients are always related to specific and discrete coronary stenosis.

We demonstrated that mortality was low, and that severe coronary stenosis was not associated with increased long-term mortality in our population with normal MPI. Importantly, the majority of these patients were prescribed aspirin and statins. This confirms the generally good prognosis of stable coronary disease, with an annual incidence of cardiac death and non-fatal myocardial infarction between 0.6 and 1.4% and 0.6 and 2.7%, respectively [23, 24].

In patients with persistent symptoms suggestive of angina and recent normal non-invasive functional test results an initial trial of optimal medical treatment combined with lifestyle interventions should be considered. When symptoms don’t improve despite medical treatment, further intensification of medical treatment is recommended. If symptoms persist, ICA with FFR measurement should be considered as recommended in the ESC guideline [8]. In patients with a high pre-test likelihood of coronary artery disease false negative SPECT results may occur as Bayes’ theorem predicts that a high post-test likelihood will remain as well. However, several studies have demonstrated that current pre-test likelihood scores overestimate the true prevalence of obstructive stenosis found in patients with typical angina [25]. Therefore, non-invasive coronary computed tomography angiography could be used as an alternative to exclude the presence of relevant obstructive CAD following a normal SPECT scan in patients with persisting symptoms [26]. Other alternatives to depict false negative SPECT may include quantitative positron emission tomography MPI [27] or cardiac perfusion magnetic resonance imaging (MRI) [28].

Our study has several limitations. First, we studied a highly selected population, who had ICA after normal MPI because of persisting symptoms, and had no data on patients without subsequent ICA after normal MPI. Second, the data were retrospectively collected. Hence, misclassification of baseline characteristics may have occurred. Third, FFR was performed in only few patients, and the haemodynamic importance of stenosis can be discussed, particularly in our selected patients with normal MPI. However, European guidelines at the time of ICA recommended additional FFR only in patients with intermediate coronary stenosis (i. e. 50–70%), while coronary revascularisation could be considered, based on symptoms, for patients with persisting angina unresponsive to optimal medical treatment [29, 30]. In 2009, Tonino et al. showed the superiority of FFR over ICA in patients with multivessel disease [31]. Since then, we have been using FFR at our hospital more often to assess the severity of a stenosis, particularly in patients without documented ischaemia. Fourth, intramural plaques cannot be visualised by normal ICA, and neither intravascular ultrasound nor optical coherence tomography was used in our study. Fifth, further non-invasive cardiac evaluation by means of either PET imaging or cardiac MRI was not performed, but these investigations are routinely used in only a few hospitals. Finally, the majority of our myocardial perfusion procedures were performed without computed tomography-based attenuation correction. However, computed tomography-based attenuation, may possibly only improve specificity of MPI in specific subgroups [32].

## Conclusion

Increasing age, male gender, previous PCI and typical symptoms are predictors of severe stenosis at invasive coronary angiography in patients with persisting symptoms after normal nuclear MPI. The majority of these patients have single-vessel disease (not the left main coronary artery).

### Take home message

- Age, male gender, prior percutaneous coronary intervention and typical angina are predictors of obstructive coronary artery disease in patients with persisting symptoms and normal single photon emission computed tomography
- The majority of these patients have single-vessel disease
- Severe coronary stenosis was not associated with increased mortality at long-term follow-up
